# Adaptive plasticity in the gametocyte conversion rate of malaria parasites

**DOI:** 10.1371/journal.ppat.1007371

**Published:** 2018-11-14

**Authors:** Petra Schneider, Megan A. Greischar, Philip L. G. Birget, Charlotte Repton, Nicole Mideo, Sarah E. Reece

**Affiliations:** 1 Institutes of Evolution, Immunology and Infection Research, School of Biological Sciences, University of Edinburgh, Edinburgh, United Kingdom; 2 Department of Ecology & Evolutionary Biology, University of Toronto, Toronto, Ontario, Canada; University of Nottingham, UK, UNITED KINGDOM

## Abstract

Sexually reproducing parasites, such as malaria parasites, experience a trade-off between the allocation of resources to asexual replication and the production of sexual forms. Allocation by malaria parasites to sexual forms (the conversion rate) is variable but the evolutionary drivers of this plasticity are poorly understood. We use evolutionary theory for life histories to combine a mathematical model and experiments to reveal that parasites adjust conversion rate according to the dynamics of asexual densities in the blood of the host. Our model predicts the direction of change in conversion rates that returns the greatest fitness after perturbation of asexual densities by different doses of antimalarial drugs. The loss of a high proportion of asexuals is predicted to elicit increased conversion (terminal investment), while smaller losses are managed by reducing conversion (reproductive restraint) to facilitate within-host survival and future transmission. This non-linear pattern of allocation is consistent with adaptive reproductive strategies observed in multicellular organisms. We then empirically estimate conversion rates of the rodent malaria parasite *Plasmodium chabaudi* in response to the killing of asexual stages by different doses of antimalarial drugs and forecast the short-term fitness consequences of these responses. Our data reveal the predicted non-linear pattern, and this is further supported by analyses of previous experiments that perturb asexual stage densities using drugs or within-host competition, across multiple parasite genotypes. Whilst conversion rates, across all datasets, are most strongly influenced by changes in asexual density, parasites also modulate conversion according to the availability of red blood cell resources. In summary, increasing conversion maximises short-term transmission and reducing conversion facilitates in-host survival and thus, future transmission. Understanding patterns of parasite allocation to reproduction matters because within-host replication is responsible for disease symptoms and between-host transmission determines disease spread.

## Author summary

Malaria parasites in the host replicate asexually and, during each replication cycle, some asexuals transform into sexual stages that enable between-host transmission. It is not understood why the rate of conversion to sexual stages varies during infections despite its importance for the severity and spread of the disease. We combined a mathematical model and experiments to show that parasites adjust conversion rates depending on changes in their in-host population size. When population sizes plummet, between-host transmission is prioritised. However, smaller losses in number elicit reproductive restraint, which facilitates in-host survival and future transmission. We show that increased and decreased conversion in response to a range of in-host environments are actually part of one continuum: a sophisticated reproductive strategy similar to that of multicellular organisms.

## Introduction

Life history theory, developed for multicellular organisms, predicts how organisms should divide their resources between reproduction and growth or maintenance during their lifetime [1, 2]. Unicellular malaria parasites (*Plasmodium*) also face this life history trade-off, since they use different stages for within-host survival and between-host transmission [3]. Malaria parasites replicate asexually in the blood of a vertebrate host and, during every replication cycle, a proportion of asexual stages commit to producing sexual stages (“gametocytes”) [4, 5]. Asexual stages are required for within-host survival and the parasites’ capacity for rapid asexual replication is responsible for the symptoms and severity of malaria. A round of sexual reproduction must occur in the mosquito vector making gametocytes essential for between-host transmission. Across *Plasmodium* species, allocation to gametocytes versus asexual stages (the “conversion rate”) is generally low but highly variable during—and between—infections [6–8]. Explaining low but variable conversion rates is a long-standing challenge in parasitology [9–12] and understanding plasticity in reproductive allocation is a major aim of evolutionary biology [1, 2].

Variation in conversion rates represents a form of “phenotypic plasticity” in reproductive allocation [3], which is commonly observed in multicellular taxa [1, 2]. In its broadest sense, phenotypic plasticity is defined as a single genotype producing different phenotypes across environments [13]. We apply the term here to refer to changes in phenotype (conversion rate) in response to directional shifts in the environment. This contrasts with “bet hedging”, which refers to the production of multiple phenotypes independent of the direction of environmental change. Phenotypic plasticity can be “adaptive”, allowing organisms to maximise fitness in a particular environment by matching trait values to the conditions they encounter [13].

Life history theory provides intuition to explain plasticity in reproductive allocation: organisms in good physiological condition (“state”) and/or with plentiful resources can afford to invest in reproduction, but as their physiological condition deteriorates (e.g. due to ageing or a deteriorating environment) they should divert resources away from reproduction and into maintenance and survival—a strategy called “reproductive restraint” [14–18]. This maximises fitness by increasing the likelihood of survival until the environment or state improves, consequently increasing the likelihood of future reproduction. However, if survival is extremely unlikely and prospects for future reproduction are bleak, organisms should prioritise allocation of resources to reproduction—a strategy called “terminal investment” [1, 2, 17–20]. Estimating how and why a trait of a given genotype changes across a range of possible states or environmental conditions (i.e. the “reaction norm”) is difficult. Since environmental conditions can vary in many ways simultaneously, it is rarely obvious which specific conditions trigger changes in allocation to reproduction. Further, comparing fitness to what would be expected if the trait were not plastic or adjusted to other values is needed to determine whether plasticity is adaptive. Finally, identifying terminal investment can be particularly difficult since being in poor state means that an organism’s absolute level of reproductive output may be low, despite investing all they can.

The concepts of state, reproductive restraint, and terminal investment are readily applied to malaria. When viewing asexual stages of a given genotype within a host as an individual multicellular organism that can grow and reproduce [21, 22], conversion rate is analogous to reproductive investment. Reasonable proxies for state (a notoriously vague term) include the density, or changes in density, of the cohorts of asexual stages that make conversion rate ‘decisions’ [3]. As a product of infections taking their natural course, or due to interventions such as drug treatment, parasites experience rapid and extensive variation in the within-host environment and so too, their state. A wealth of data, collected from human parasites *in vitro* and animal models, suggest that differences in the density and age structure of red blood cell resources, the presence of co-infecting parasite strains, and antimalarial drug treatments can all alter conversion rates [23–43]. We and others have proposed that this apparent plasticity in the conversion rate is a life history strategy adapted to coping with the changeable ecology malaria parasites experience during infections (reviewed in [3, 8]).

**Fig 1:**
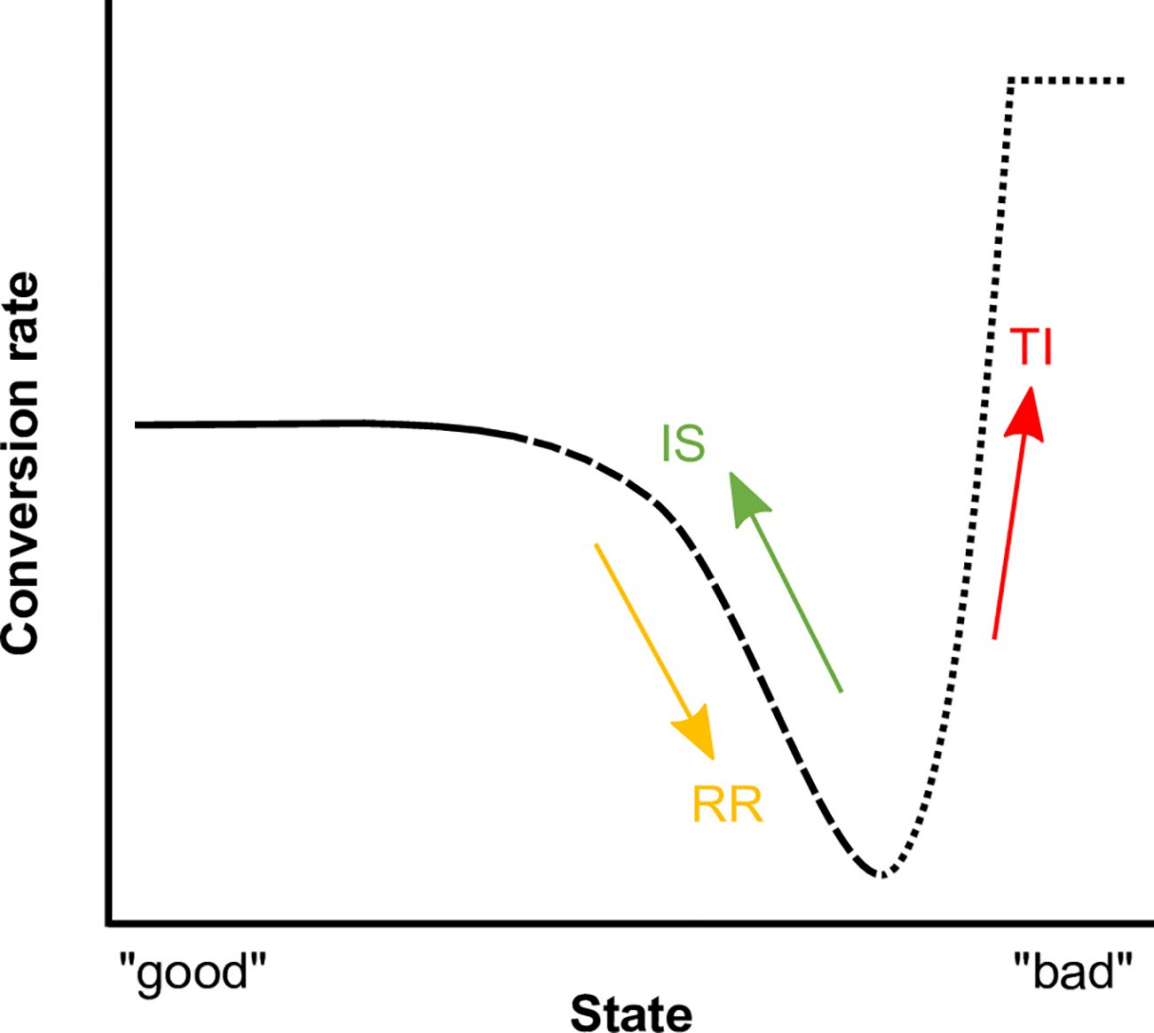
Predicted reaction norm for plasticity in gametocyte conversion rate in response to perturbations of state. State refers to a combination of physiological condition and environmental factors. Evolutionary theory for life histories as applied to malaria parasites suggests that genotypes in good state can afford to invest in gametocytes (solid line), but should adopt reproductive restraint if state deteriorates (dashed line), and switch to making a terminal investment (dotted line) if state deteriorates so much that in-host survival becomes unlikely. Note that the x axis is not synonymous with time post infection and that parasite state may improve and deteriorate multiple times during an infection. Most studies in Table 1 can be qualitatively interpreted according to this pattern.

Why might malaria parasites benefit from altering conversion in response to state? For example, early in infections, while parasite densities are growing exponentially, parasites exhibit good state, whereas attack by antimalarial drugs or immune responses causes a decline in state. When parasites experience a low to moderate loss of state, reproductive restraint could facilitate within-host survival and future transmission by diverting investment to asexual replication, albeit at a cost to short-term transmission. In contrast, when in-host survival is unlikely due to severe loss of state, switching to terminal investment might maximise short-term transmission. Thus, intuitively, conversion rate should follow a non-linear reaction norm with respect to declining parasite state (Fig 1). Whereas many studies report responses in the direction that our intuition predicts [23–52] (Table 1), some predict no change in conversion rate despite a change in state [23, 45, 53], while others reveal different responses to the same perturbation (e.g., drug treatment [24, 37]). Unfortunately, existing experimental data preclude building a reaction norm, due to several issues. First, previous approaches used to estimate conversion rates are likely to be biased [54]: when even a fraction of gametocytes persist longer than the sampling interval, conversion rates can be wildly overestimated. This bias is exacerbated when asexual densities are declining (as would be the case for any perturbation resulting in a loss of state). Second, many studies compare only two groups, i.e. a single perturbation and a control, providing limited resolution on the full breadth of the reaction norm.

**Table 1:** Studies on plasticity in *P*. *chabaudi* and *P*. *falciparum* conversion rates following a variety of perturbations that affect state.

**NC. No change: deteriorating state associated with no change in conversion rates**		
*P*. *chabaudi in vivo*	[53]	competition
*P*. *falciparum in vivo*	[45]	drugs
*P*. *falciparum in vitro*	[23]	drugs
**IS. Improved state associated with increased conversion rates**		
*P*. *chabaudi in vivo*	[26–28,31]	reticulocytes
*P*. *falciparum in vitro*	[23,29–30]	reticulocytes
*P*. *falciparum in vivo*	[44,46,51–52]	anaemia/reticulocytes
**RR. Reproductive restraint: deteriorating state associated with decreased conversion rates**		
*P*. *chabaudi in vivo*	[25, 31–33]	competition, drugs, high parasite density, lysed infected RBC
*P*. *falciparum in vitro*	[23–24,34–35]	drugs, co-culture healthy parasites
*P*. *falciparum in vivo*	[49]	drugs
**TI. Terminal Investment: deteriorating state associated with increased conversion rates**		
*P*. *chabaudi in vivo*	[36,38–39]	drugs/ immunity
*P*. *falciparum in vitro*	[23,35,37,40–43]	drugs/immunity/conditioned medium/co-culture crisis parasites
*P*. *falciparum in vivo*	[45–50]	drugs

Results fall within 4 categories of which 3 are consistent with the reaction norm predicted in Fig 1. The group that appears inconsistent with the reaction norm (NC) shows no change in conversion rate in response to perturbations of state. It is possible that these studies have observed 2 points on the reaction norm where conversion is coincidentally the same, for example, conversion is high when state is good and during terminal investment. These studies span different species of parasite, experimental approaches, types of perturbations, and the accuracy of methods used to estimate conversion rate, making our interpretations qualitative. Some studies feature in multiple categories if for example, different parasite genotypes respond differently or different drugs elicit different responses. Note that Carter and Miller [35] is of particular relevance, illustrating medium, low and high conversion in *P*. *falciparum in vitro* cultures with good, deteriorating and bad state, respectively, though the authors did not interpret their data in that way.

Here, we overcome the above issues by combining a new method for inferring conversion rates [54] with experiments using different doses of an antimalarial drug as a convenient way to perturb parasite state in a quantifiable manner. We first use a mathematical model to test the intuition that adaptive conversion rates follow a non-linear reaction norm in response to drug-induced perturbations of parasite state. We then use the rodent malaria *Plasmodium chabaudi* to test these expectations, estimating conversion rates across the entire reaction norm (from no change in state due to drugs to near clearance of infections), and more accurately than previously possible [54]. We then assess how the observed changes in conversion rates impact upon prospects for within-host survival and short-term transmission. Further analyses show that whilst state is a major determinant of conversion rate, parasites also fine-tune conversion decisions in response to the dynamics of red blood cell resources. We then revisit data from previous experiments using different parasite genotypes and perturbations of state to test the generality of our results. Elucidating the shape of the reaction norm for conversion rates in response to changes in state is important because many disease control measures, like drug treatment, seek to alter parasite state. If, in response, parasites reduce conversion, infections may become harder to clear, while an increase in conversion can enhance short-term transmission. Understanding when each of these responses is expected is therefore important for mitigating the clinical and epidemiological consequences of adaptive plasticity in conversion rates.

## Results and discussion

### Plastic conversion rates maximise fitness *in silico*

Here we examine whether drug treatment can generate a non-linear reaction norm that includes reproductive restraint and terminal investment. We use a mathematical model of within-host infection dynamics that tracks the changes in densities of infected and uninfected red blood cells, as well as gametocytes, to predict the patterns of reproductive allocation (conversion rate) that maximise “fitness” in response to variation in “state” (see ‘Mathematical model’ in Materials and methods). The mathematical model perturbs state by simulating the killing of asexually replicating stages by different doses of pyrimethamine treatment (administered on day 11 post infection) of single-genotype infections of the rodent malaria *P*. *chabaudi* (S1 Fig). We assume a pattern of conversion that is predicted to be optimal in untreated single genotype infections [55, 56], then use optimization algorithms to predict the conversion rates that maximise parasite fitness following drug treatment. In essence, this model predicts shifts in conversion rates due to different losses of state that can be qualitatively compared to experimental data. This differs from our previous modelling work, which allows parasites to ‘prepare’ in advance of drug treatment [56].

**Fig 2:**
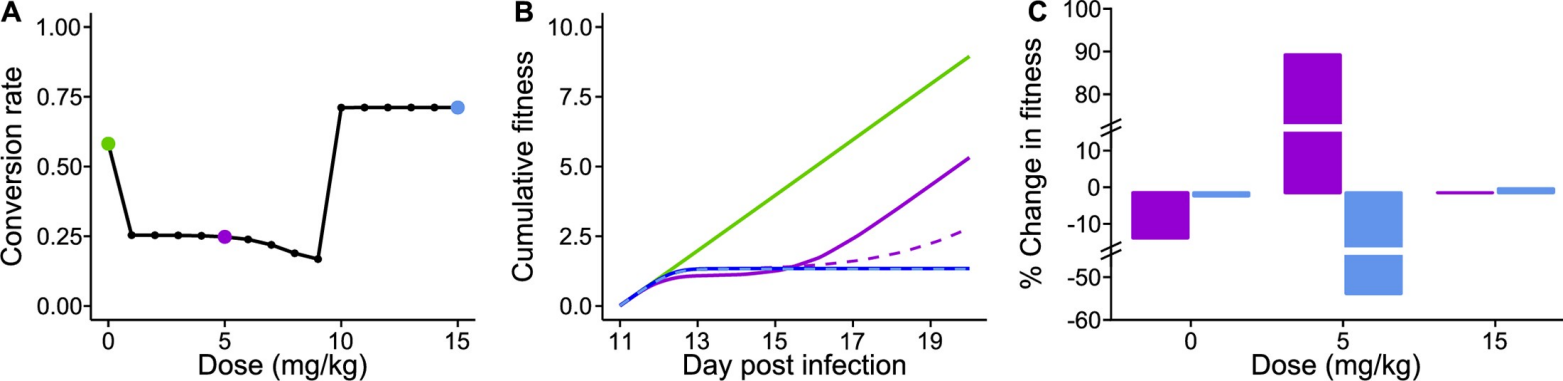
Conversion rate is adaptively adjusted *in silico* in response to a loss of state in a non-linear manner. Predicted optimal strategies and their fitness consequences across drug doses that reduce parasite state; strategies highlighted for untreated infections (green: 0 mg/kg), low dose (purple: 5 mg/kg) and high dose (blue: 15 mg/kg) drug treatment. (*A*) Compared to untreated infections, parasites should reduce conversion in response to low doses (reproductive restraint) but increase conversion in response to high doses (terminal investment). (*B*) Fitness, measured as cumulative transmission potential post-drug treatment, is highest in untreated infections (green) and reduced by drug treatment (purple and blue). Altering conversion rates allows parasites to partially compensate for the fitness costs of drugs. When treated with a low dose, reproductive restraint (solid purple line) allows parasites to recover to the same rate of transmission as untreated infections (as indicated by the slope of the solid green and purple lines) within 6 days. This also yields greater fitness than if parasites do not adjust conversion and follow the optimal strategy for untreated infections (dashed purple line). Although too small to visualise due to the scale of y-axis, changing conversion in response to a high dose yields a 1% fitness benefit (solid blue line) compared to parasites that do not adjust conversion and follow the optimal strategy for untreated infections (dashed blue line). (C) Optimal strategies are dose-specific. Fitness is calculated for parasites that follow the optimal strategies for low (purple) or high (blue) doses in each of the untreated, low dose, and high dose environments, relative to the optimal strategy for untreated infections. Adopting reproductive restraint (purple) or terminal investment (blue) costs fitness in untreated infections. At low doses, reproductive restraint (purple) is the optimal strategy and terminal investment is the poorest strategy. At high doses, terminal investment (blue) is marginally better than reproductive restraint (purple).

We find that, compared to untreated infections, parasites should reduce their conversion when confronted with low drug doses but increase conversion at higher doses (Fig 2*A*), as expected. Adjusting conversion rates partially compensates for the fitness lost due to drug treatment (Fig 2*B*), and dose-specific strategies maximise fitness (Fig 2*C*) (for calculations of fitness consequences see ‘Mathematical model’ in Materials and methods). Our model predicts a hard switch from reducing to increasing conversion once drug doses are high enough to kill >99.9% of parasites (~9 mg/kg). In reality, in addition to the challenges of accurately estimating state, parasites *in vivo* will face other factors simultaneously affecting state (e.g. host immunity) that are not included in our model. Notwithstanding these caveats, the model predicts a non-linear reaction norm because parasites that plastically adjust conversion in response to drugs are fitter than parasites which do not respond.

### Adaptive conversion rate modulation *in vivo*

To quantify the reaction norm for conversion rates in response to drug treatment, we established *P*. *chabaudi* (genotype ER) infections in laboratory mouse hosts and treated parasites at 2pm GMT on day 11 post infection (PI) with different doses of pyrimethamine, which kills asexual stages but not gametocytes [36] (see ‘Experiments’ in Materials and methods). Pyrimethamine does not directly kill asexuals but accumulates inside them and prevents them from reaching the end of the asexual cycle. Commitment to the production of asexual- or sexual-stage progeny is thought to occur in the latter part of the asexual cycle [40, 57–60] so our treatment was timed to affect the conversion decision of the treated cohort. We focus on day 11 because even after drug treatment, the densities of asexuals and the resulting gametocytes are high enough to quantify (i.e. exceed the detection thresholds of our assays) regardless of whether parasites increase or decrease conversion (for asexual parasite and gametocyte dynamics see S2 Fig). For each infection, we examine whether the conversion rate produced by the parasites that survive to the end of the asexual cycle on day 11 PI correlates with the change in asexual density from day 11 to 12 PI. We quantified conversion rates using a new method for statistical inference that overcomes the limitations of applying previous approaches to dynamic infections [54]. This method uses longitudinal infection dynamics to estimate the conversion rates adopted on each day PI for the duration of each infection. In particular, it takes into account the accumulation of gametocytes over time, which is especially important when asexual densities are in decline, making the method uniquely suited to testing parasite responses to drug treatment [54] (see ‘Calculating conversion rate’ in S1 Text).

**Fig 3:**
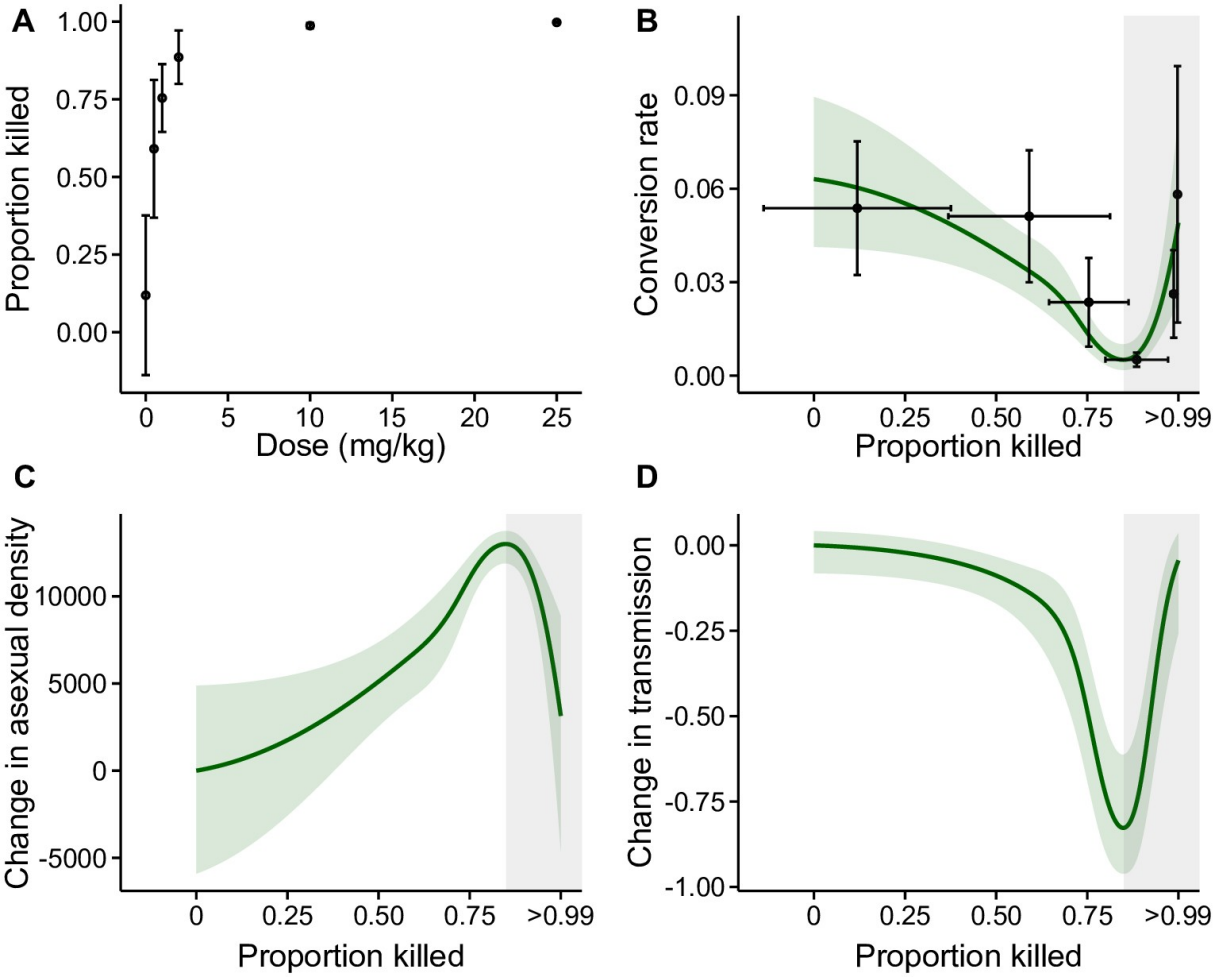
*P*. *chabaudi* parasites *in vivo* adjust conversion rate in response to a loss of state in a non-linear manner. (*A*) Dose-response curve for the proportional change in asexual density (“state”) after drug treatment. Note, at the highest drug doses, >99% but <100% of asexuals are lost and because infections were treated post peak, densities also decline in the untreated control infections. (*B*) Parasites reduce conversion in response to losing up to 85% of their number (reproductive restraint) and then switch to increasing conversion (terminal investment; grey shaded area). Following [54], conversion rate is estimated for each infection based on gametocyte, asexual and RBC dynamics (see S2 Fig). Mean ±SEM conversion rates are presented in black. The observed conversion rate strategies have short-term fitness consequences in terms of within-host survival (*C*) and between host transmission (*D*). Specifically, relative to untreated infections, reproductive restraint increases replication rate (*C*) but reduces the estimated probability of between-host transmission (translated from gametocyte densities according to [61]) (*D*). All green lines and green shaded areas are the predicted relationships ±SEM between parasite strategies and the loss of state from a generalised additive model, and grey shaded areas represent the region in which parasites make a terminal investment.

At the time of treatment, asexual stage densities were 2.24 (± SEM 0.06) *10^5^ / μL blood. Compared to pre-treatment densities, a loss of between 59 and >99% of parasites (Fig 3*A*) occurred by day 12 PI due to the net effect of dose-dependent drug killing and replication of the survivors. Neither red blood cell (RBC; *F*_5,33_ = 0.258, *P* = 0.93), asexual stage (*F*_5,33_ = 0.917, *P* = 0.48), nor gametocyte densities (*F*_5,33_ = 0.859, *P* = 0.52) at the time of treatment differed significantly across treatment groups (S2 Fig). Parasites adjusted conversion rates according to the proportion of asexual stages lost from day 11 to 12 PI, in a nonlinear manner (*P* < 0.01, Fig 3*B*). Parasites that experienced a reduction of up to 85% of asexual stages decreased conversion, consistent with reproductive restraint, whereas conversion rates increased when more than 85% were lost (shaded area in Fig 3*B*), consistent with terminal investment.

The observed strategies, which cannot be explained by differential mortality of gametocytes and asexuals, are consistent with those adopted by multicellular taxa and those predicted to maximise fitness. However, observing these strategies does not directly reveal the fitness consequences of adjusting conversion. We therefore estimated the impacts (see ‘Data analysis’ in Materials and methods) of changing conversion, compared to not changing conversion, on within-host replication (Fig 3*C*) and the probability of between-host transmission (translated from gametocyte densities according to [61]) (Fig 3*D*). Reproductive restraint increases within-host replication rate (Fig 3*C*) but incurs a short-term cost to transmission (Fig 3*D*), whereas terminal investment enhances short term transmission (Fig 3*D*) at a cost to replication (Fig 3*C*).

Overall, our data qualitatively confirm the predictions of our mathematical model—when malaria parasites experience a change in state they plastically adjust conversion rates to maximise fitness by either decreasing or increasing conversion depending on the magnitude of the assault on state. Previous experimental tests have probed parts of the reaction norm and suggested reproductive restraint and terminal investment, but few studies—mainly in insects—have quantified reproductive investment across the entire reaction norm [62–64]. Although terminal investment is defined as the allocation of all remaining resources to reproduction, even the most severe loss of asexuals in our experiment did not result in all asexuals producing gametocytes. Parasites may experience constraints analogous to those operating in multicellular organisms at the end of life, where little energy is available to allocate to survival or reproduction when health is deteriorating and so, even when ‘terminal’, actual levels of investment are often low [17, 62, 65]. Alternatively, the mechanism by which parasites make a terminal investment may simply be the prevention of reproductive restraint.

### State sensitive conversion rates across genotypes

Having found support for a non-linear reaction norm, we now ask what information parasites base their conversion rate decision on. We propose that parasites adjust conversion rates in response to a change in state, rather than detecting the concentration of pyrimethamine they are confronted with. This is because the *P*. *chabaudi* genotype we used has not previously been exposed to antimalarial drugs, drug resistant *P*. *falciparum* isolates do not adjust conversion in response to drugs [24], and changes in conversion in both species have been recorded in response to a range of drugs (Table 1). Moreover, increases and decreases in conversion can be elicited by the same drug [24, 37]. In the previous section, we focused on the proportional reduction in asexual stages as a proxy for a change in state (Fig 3*B*) but the absolute number of asexual stages lost may also be a good proxy for state. While the absolute number lost produces a qualitatively similar pattern for conversion rates (*P* = 0.01, S3 Fig), it explains significantly less variation than the proportional reduction (S1 Table). This suggests that asexuals either monitor the proportion of their cohort that will survive to the end of the cycle or measure a proxy that correlates more closely with proportional change than the actual number that will die. Intuitively, monitoring a change is a more informative metric for state than absolute number: density alone does not allow parasites to differentiate between increasing or decreasing densities. For example, low density may be due to imminent clearance or because parasites are establishing a new infection.

To further examine whether parasites respond to variation in their state we analyse previously published data from two independent experiments (see ‘Experiments’ in Materials and methods) for which conversion rates can be estimated using Greischar et al’s method [54]. The first experiment [66], exposed *P*. *chabaudi* genotype CWvir parasites to different doses of pyrimethamine on day 5 PI to generate a range of asexual densities by days 11–15 PI. The second experiment [25], perturbs asexual densities of genotypes AS and AJ via within-host competition with another genetically distinct genotype. These datasets include both expanding and declining infections. Proportion killed is not an applicable measure of state for expanding infections so, here, we use replication rate (from the conversion-decision-making cohort to its progeny) as a proxy for state. We examine conversion rates for CWvir during days 11–15 PI and on day 9 PI for AS and AJ because these periods reflect when parasites in control infections have conversion rates at intermediate levels, which facilitates the observation of both increased and decreased conversion.

**Fig 4:**
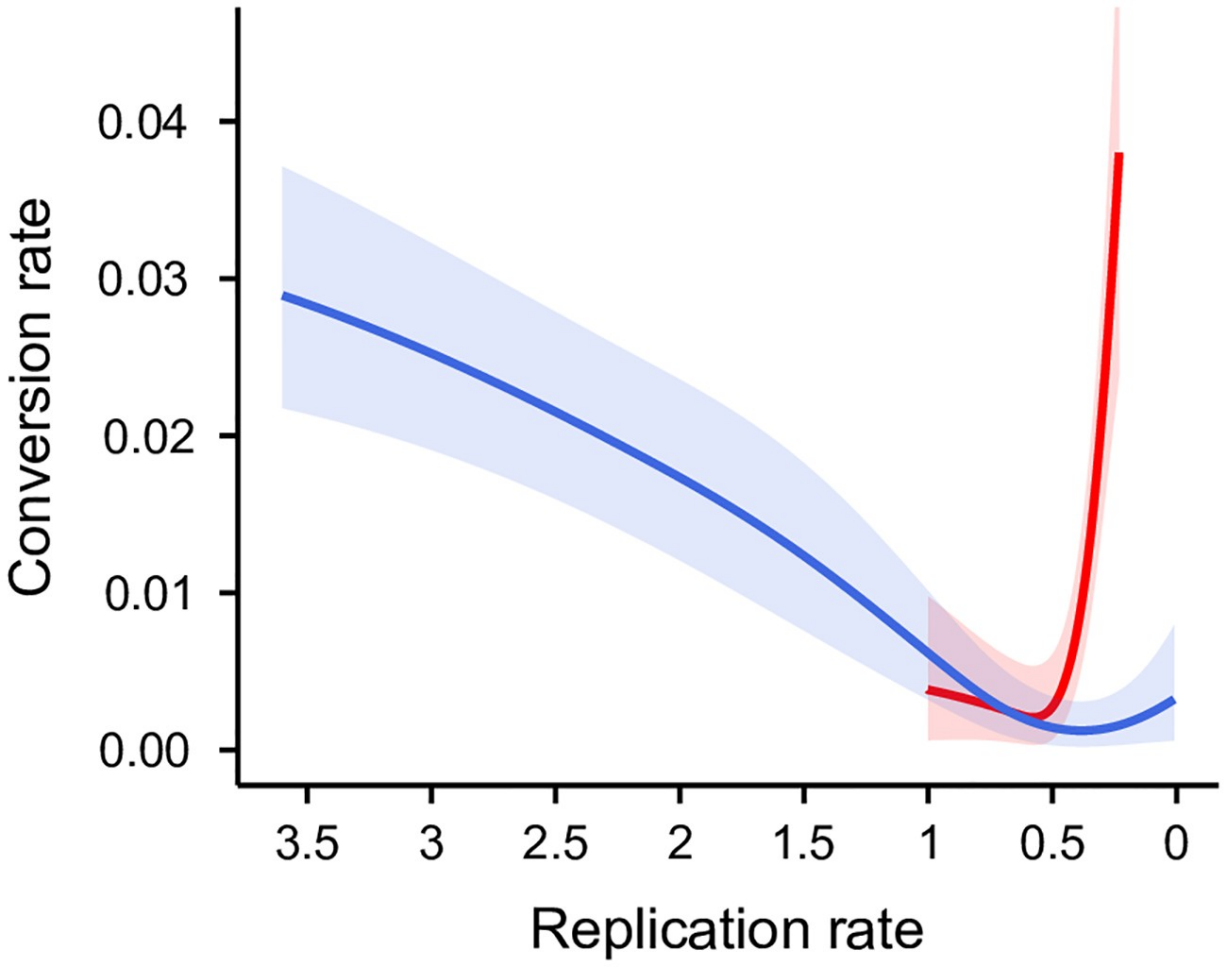
Other *P*. *chabaudi* genotypes adjust conversion in response changes in state. The densities of asexual stages were perturbed by drug treatment for genotype CWvir (blue) and by within-host competition with one or two other *P*. *chabaudi* genotypes for AS and AJ (combined, red). For these data we use replication rate as a proxy for state, calculated as the density of the asexual cohort making the conversion rate decision relative to the density of its asexual progeny. Replication rates are >1 for expanding infections and <1 in declining infections. Following [25, 66], conversion rate is estimated for each infection based on gametocyte, asexual and RBC dynamics. Solid lines and shaded areas are predictions ± SEM from generalised additive models for conversion as a function of replication rate.

For all genotypes we find that parasites invest in gametocytes when asexual replication is exponential, reduce conversion as replication becomes constrained, and increase conversion when infections decline severely (CWvir *P* < 0.01; AS and AJ *P* = 0.04; Fig 4). Thus, as we find for genotype ER in our main experiment (Fig 3*B*), the reaction norms for CWvir, AS and AJ switch from reproductive restraint to terminal investment as state declines. For ER, the switch point between reproductive restraint and terminal investment is predicted when 85% of asexual stages are lost, compared to 43% for CWvir, and 61% for AJ and AS combined. These differences may reflect inter-experiment variation or genetic variation for how sensitive conversion rates are to changes in state (i.e. a dynamic threshold for terminal investment [14]). Different sensitivities to state could relate to differences in virulence, which affects the capacity to recover replication. Further work to investigate reaction norms in relation to virulence is required to test whether more virulent genotypes can withstand greater loss in number before making a terminal investment or make a terminal investment early to compensate for the elevated risk of host death.

Finally, the data for AS and AJ have been used previously to show that within-host competition induces reproductive restraint [25]. Here, we find that changes in state can equally explain the effect of competition (including competition treatment does not increase the explanatory power of statistical models with state; *P* = 0.10). Because state is the integrated effect of all within-host factors that impact on asexual replication, monitoring state may enable parasites to respond adaptively to a diversity of within-host factors without the need to detect each of them individually. Thus, we envisage that plasticity in conversion has been selected for by within-host stressors such as immune responses, competition, and resource limitation rather than as a specific adaptation to cope with drug treatment; since parasites respond to state, this enables them to mount an appropriate response to drugs too.

### Red blood cell resource availability impacts on conversion

The adjusted r^2^ values for the reaction norms capturing variation in measures of state and conversion rate for all three experiments range from 0.33 to 0.47, suggesting that additional factors influence conversion rates. In addition to asexual stages acting as a resource to be invested into gametocytes, RBC are another essential resource. Indeed, there are associations between the development of anemia and gametocyte densities in natural infections of humans, and the age structure of RBC strongly influences conversion rates of *P*. *chabaudi* [26, 27, 52, 67]. The availability of RBC also shape the state-dependent decisions *P*. *chabaudi* makes regarding the ratio of male to female gametocytes [68]. Therefore, we investigated whether, in addition to state (using the proportion of asexual stages lost as a proxy), variation in the availability of RBC also influences the conversion rates of genotype ER in our main experiment.

**Fig 5:**
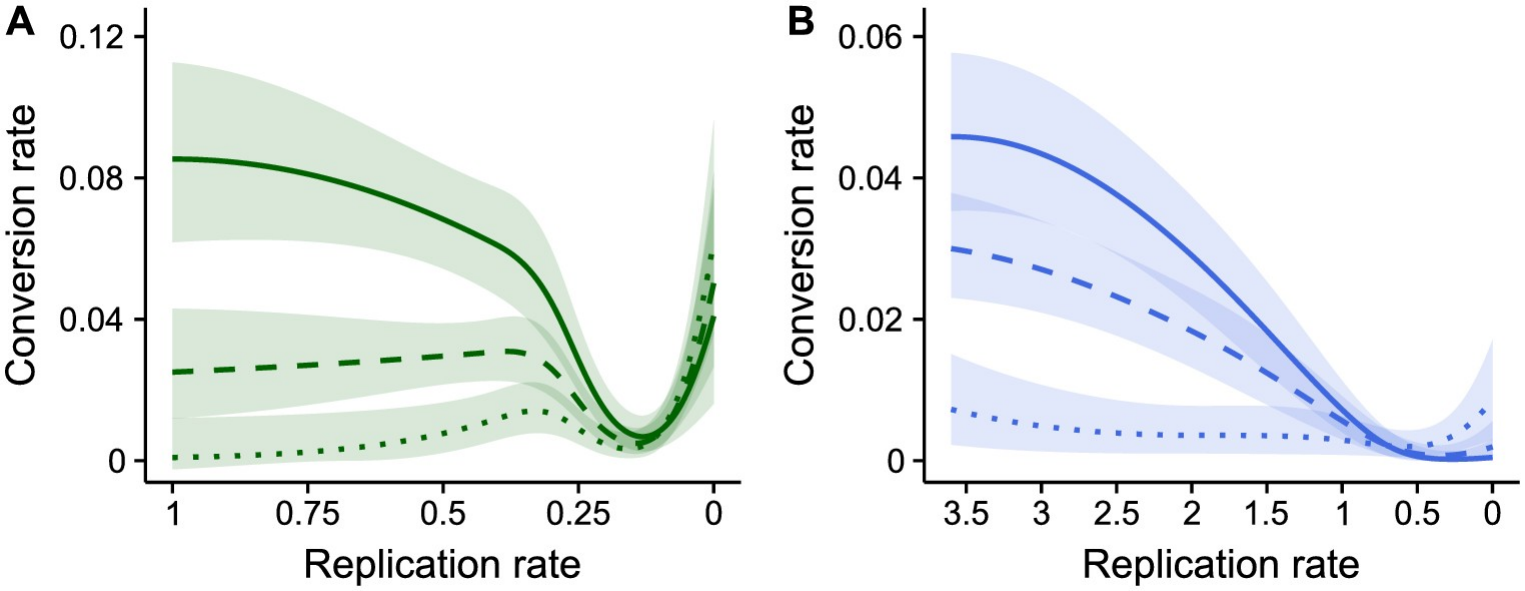
In addition to state, changes in red blood cell (RBC) density modulate conversion rates. The reproductive restraint region of reaction norms for (*A*) ER and (*B*) CWvir are steeper when RBC density is increasing (solid lines; 90^th^ percentile) compared to remaining constant (dashed lines; 50^th^ percentile) or decreasing (dotted lines; 10^th^ percentile). Percentiles for RBC change are used to normalise for the differing dynamics between the experiments and are calculated as the difference in RBC density between the decision-making and parental cohorts of asexuals. Solid lines and shaded areas are predictions ± SEM from generalised additive models for conversion as a function of both replication rate (state) and changes in RBC density.

We compared three generalised additive models with the following RBC metrics fitted as main effects and in interactions with state: (1) The absolute density of RBC midway through the cycle of the decision-making cohort. (2) The difference between RBC densities experienced by the decision-making cohort and its parental cohort. (3) The absolute density of RBC midway through the cycle of the parental cohort. Statistical models (1) and (3) reflect the possibility that parasites are sensitive to resource abundance *per se*, either during the period of commitment to asexual or sexual progeny (1) or during invasion of RBC at the beginning of the cycle (3); whereas statistical model (2) asks if parasites are sensitive to whether resources are increasing or decreasing. The best statistical model (determined by AIC; S2 Table) is model (2), with an adjusted r^2^ of 0.61. State retains the strongest association with conversion rates, but when RBC density is increasing, parasites are more likely to adopt reproductive restraint in response to a loss of state (Fig 5*A*). The data for CWvir [66] reveal a similar reaction norm: parasites with increasing RBC have higher conversion when in good state and are more likely to adopt reproductive restraint when state deteriorates (adjusted r^2^ = 0.55, *P* < 0.01, Fig 5*B*). Note, the data set for AS and AJ [25] has insufficient variation in RBC densities for this analysis. The results for ER and CWvir suggest that parasites adopt stronger reproductive restraint when hosts are recovering from anaemia. Prioritizing within-host replication may enable parasites to take advantage of incoming RBC and maximise the source population of asexuals for future investment into gametocytes.

**Fig 6:**
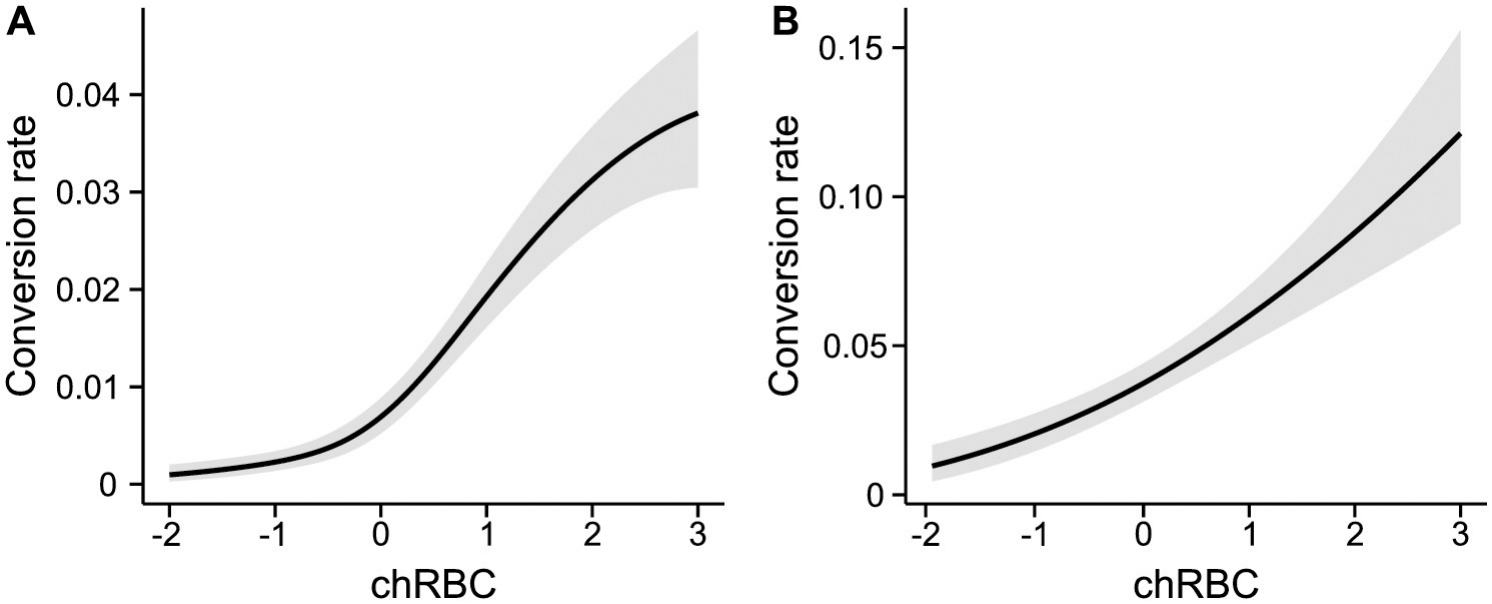
Parasites in good state increase conversion rates as RBC densities increase. RBC density was perturbed independently of parasite state for *P*. *chabaudi* parasites exposed to EPO (*A*, days 4–14 PI) or phenylhydrazine (*B*, day 1 PI). Change in RBC refers to the difference in RBC density between the decision-making and parental cohorts of asexuals. Parasites were in good state during treatments in both experiments, with >90% exceeding a replication rate of 1.61 (*A*) or 3.3 (*B*). Note, conversion in (*A*) is estimated following [54], while a simpler metric had to be used for (*B*) (see ‘Experiments’ in Materials and methods). Lines and shaded areas are predictions ± SEM from generalised additive models for conversion as a function of changes in RBC density.

Our analyses suggest that parasite responses to changes in state are modified by the availability of RBC resources, such that parasites in good state adopt higher conversion rates when RBCs are increasing compared to when RBC are decreasing. To explore this further we analysed two additional previous experiments in which: (i) ER infected hosts were treated with erythropoietin (EPO) during days 4–7 PI [26] and (ii) hosts were treated with phenylhydrazine (PHZ) prior to infection with genotype ER, AS, AJ, or CR [27]. These experiments allow us to investigate parasite responses to a range of changes in RBC density in the absence of confounding perturbations in state. Although EPO stimulates the release of reticulocytes into the blood, both EPO-treated and control hosts experienced the same range of changes in RBC densities during the period we examine conversion rates (days 4–14 PI). As opposed to EPO treatment, hosts treated with PHZ experienced substantially reduced RBC density as well as an increase in the proportion of reticulocytes. We examined conversion on day 1 PI because state begins to diverge between control and PHZ-treated hosts on subsequent days. We focus on whether conversion rates correlate with the difference between RBC densities experienced by the decision-making cohort and its parental cohort (i.e. as for statistical model 2, above). Conversion rates correlate with changing RBC dynamics (adjusted r^2^ = 0.26, *P* < 0.01, Fig 6*A*) in the same manner in EPO-treated and control hosts (F_1,5_ = 0.016 p = 0.898). Specifically, conversion follows a non-linear pattern: parasites in good state, i.e. with positive replication rates, adopt higher conversion rates if RBCs are increasing. Similarly, in the PHZ experiment, conversion increases nonlinearly with increasing RBC densities (adjusted r^2^ = 0.19, *P*<0.01, Fig 6*B*).

### Concluding remarks

Here, we reveal that evolutionary theory for life histories developed for multicellular taxa also applies to unicellular parasites that experience a trade-off between reproduction and survival. Previous studies in parasitology have suggested “terminal investment” but our life-history approach has detected the full breadth of the parasites’ strategy (which we show spans from reproductive restraint to terminal investment) whilst providing a rare examination, for any organism, of plasticity in reproductive investment across an entire reaction norm.

Malaria parasites have evolved a suite of strategies for surviving within the host (e.g., antigenic variation to escape immune responses [69]). Using reproductive restraint to prioritise within-host survival and the long-term transmission opportunities this brings, in all but catastrophic circumstances, is in keeping with this. Given that the strategies we observe in *P*. *chabaudi* are analogous to those of taxonomically diverse multicellular organisms, we expect that human malaria parasites *in vivo* [44–52] (Fig 1, Table 1) also adopt these strategies in response to changes in state rather than the presence/concentration of specific drugs [24]. If so, plasticity in conversion rates has clinical and epidemiological consequences: reproductive restraint makes infections harder to clear, which could explain why some drug treated infections persist and spread in the absence of drug resistance mutations [70], while terminal investment increases short-term transmission, highlighting the value of drugs with gametocytocidal action. Further, outcomes of drug treatment may be affected by anaemia, independent of parasite dynamics [71]: if patients vary in the rate at which erythropoiesis replenishes RBC, the same drug dose may induce different degrees of reproductive restraint by parasites. Stronger reproductive restraint (to capitalise on the influx of resources) in patients recovering quickly from anaemia could delay clearance and facilitate development of chronic infections. Ideally, treatment should elicit terminal investment whilst deploying transmission-blocking measures.

Our analyses suggest the following scenario for how parasites make conversion rate decisions. Each cohort of asexual stages commits to producing gametocytes or not in the latter half of their cycle. This supports previous suggestions that commitment takes place at the schizont stage [40, 57–60]. The conversion rate is based primarily on cue(s) reflecting how the density of asexuals will change between the current and subsequent cycles, and information about the supply of RBCs further modulates conversion, especially when parasites are in a good state. How parasites detect state is unknown but recent work suggests *P*. *chabaudi* detects components of lysed asexuals in the blood [32], while *P*. *falciparum* parasites may titrate the concentrations of different signals in exosomes [72], as well as the concentration of a host-derived lipid, LysoPC, which is known to affect parasite state and conversion rate [40]. Parasites could deploy cell-cell communication to disseminate information about state or respond individually to a proxy, such that on average across the cohort, the correct conversion decision is made. The cues (or proxies) relating to RBC resources are also unknown, although the age of an RBC has been ruled out as having a direct influence on the conversion decision of the parasite developing within it [27]. The molecular mechanisms regulating the production of gametocytes are being uncovered [40, 72] so the next challenge is to broaden this mechanistic understanding to ask how parasites sense state and feed this information into commitment pathways. Trypanosomes exhibit similar state-dependent investment in transmission forms (“stumpies”) and their density-sensing pathway is known [73]. However, unlike malaria parasites, Trypanosomes are unable to modulate investment in response to the genetic diversity of their infections, suggesting that sensing and signalling pathways may be more sophisticated in malaria parasites [73]. Understanding such pathways opens up the possibility of interventions that trick parasites into making suboptimal life history decisions [3]. Challenges such as measuring conversion in natural infections to assess how much plasticity in conversion parasites display and how this impacts upon rates of between-host transmission and prospects for within-host survival, need to be overcome.

## Materials and methods

### Mathematical model

We extend a previously published mathematical model of the within-host dynamics of malaria infections [55, 56]. This model is composed of delay-differential equations, tracking the densities of uninfected red blood cells (RBCs, *R*), infected RBCS (Eq 1), extracellular asexual parasites called merozoites (*M*), and gametocytes (*G*). The changes in density of these variables over time, *t*, are given by

$$\begin{array}{l} \frac{dR\left(t\right)}{dt}=\lambda \left(1-\frac{R\left(t\right)}{K}\right)-\mu R\left(t\right)-pR\left(t\right)M\left(t\right)\\ \frac{dI\left(t\right)}{dt}=pR\left(t\right)M\left(t\right)-\left(\mu +\mu_{d}\right)I\left(t\right)-pR\left(t-\alpha \right)M\left(t-\alpha \right)S\\ \frac{dM\left(t\right)}{dt}=\left(1-c\left(t\right)\right)\beta pR\left(t-\alpha \right)M\left(t-\alpha \right)S-pR\left(t\right)M\left(t\right)-\mu_{M}M\left(t\right)\\ \frac{dG\left(t\right)}{dt}=c\left(t\right)pR\left(t-\alpha \right)M\left(t-\alpha \right)S-\mu_{G}G\left(t\right) \end{array}$$ (1)

where λ is the maximum rate of replenishing RBC, *K* is the homeostatic equilibrium density of RBC, *p* is the rate at which asexual parasites invade RBCs upon contact, and β is the number of parasites released from each infected RBC surviving the developmental period. The developmental period is given by α, and is assumed to be one day for *Plasmodium chabaudi*. Death rates of RBC, merozoites, and gametocytes are given by μ, μ_M_, and μ_G_, respectively. Drug-induced killing of infected RBCs occurs at rate μ_d_; as in previous work, we assumed that drug dose affects the period of time over which parasite killing occurs (the day of drug treatment plus *L* additional days, where *L* is dose-dependent), but not the rate of killing [56] as was previously quantified from experimental data [74]. Specifically,

$$L=3.557-\frac{2.586}{1+e^{-8.821+d}},$$ (2)

where *d* is drug dose in mg/kg [56, 74]. In this delay-differential equation model, survival of infected cells over this developmental delay must be explicitly tracked and is given by

$$S=\{\begin{array}{ll} \text{exp}\left(-\mu t\right), & t<\alpha \\ \text{exp}\left(-\left(\int_{t-\alpha }^{11}\mu d\omega +\int_{11}^{t}\mu +\mu_{d}d\omega \right)\right), & 11\leq t<\alpha +11\\ \text{exp}\left(-\left(\int_{t-\alpha }^{t}\mu +\mu_{d}d\omega \right)\right), & \alpha +11\leq t\leq 11+L\\ \text{exp}\left(-\left(\int_{t-\alpha }^{11+L}\mu +\mu_{d}d\omega +\int_{11+L}^{t}\mu d\omega \right)\right), & 11+L<t<\alpha +11+L\\ \text{exp}\left(-\mu \alpha \right), & \text{otherwise} \end{array}.$$ (3)

Finally, conversion rate is determined by *c(t)*, which is the proportion of parasites of a given cohort of infected RBCs that become gametocytes. In our current model, conversion rate was allowed to be a function of time, up until drug treatment, after which we assume that it is a constant to isolate the qualitative influence of drug treatment on conversion rates. Default parameter values are listed in S3 Table. Further details of the development of the baseline model, including a graphical description of drug action, can be found in [56]. Below we describe the extension of the model we use here.

Until day 11, *c(t)* is defined as a cubic spline with parameters given by the optimal strategy predicted for untreated infections [56] ([28, 56, 74–79] see S3 Table). This generated estimates of the densities of susceptible RBCs, infected RBCs, merozoites, and gametocytes at day 11, just before drug treatment (S3 Table; note that these densities are identical to the day 11 values plotted in black in Figure 3 of Birget et al. [56]). We then fed these values as starting conditions back into the within-host model and used the optim function in R version 3.0.2 to identify the time-constant conversion rate that maximised parasite fitness over the remainder of the infection (days 11–20), for parasites receiving either no drugs, or pyrimethamine at doses from 0 to 15 mg/kg (killing up to 99.999% of asexual stages). Following previous studies, we measure parasite fitness as the “cumulative transmission potential” (CMT) achieved by a given conversion strategy [55, 56]. This metric capitalises on the predictive value of gametocyte densities for mosquito infection [61, 80]. CMT is calculated by taking gametocyte dynamics predicted by the within-host model, translating them into daily probabilities of transmitting to mosquitoes according to one empirical estimate of this relationship for *P*. *chabaudi* [61], and summing these across the remaining days of infection as a proxy for lifetime reproductive output. From [61], and assuming all else equal with respect to other factors that could influence transmission (e.g. vector biting rates, host immunity, vector susceptibility, time-of-day of transmissions [61, 81, 82]), the probability of transmission for a given gametocyte density, *G*(*t*) is given by

$$\frac{\text{exp}\;\left[-12.69+3.6\;{\text{log}}_{10}G\left(t\right)\right]}{1+\text{exp}[-12.69+3.6\;{\text{log}}_{10}G\left(t\right)]}$$ (4)

Finally, to provide validation that the predicted ‘optimal’ strategies for a given drug environment outperform other strategies, we simulated infections with parasites employing each of the putative best strategies for representative doses of 0, 5 or 15 mg/kg, treated with each of these three drug doses (S1 Fig) and calculated CMT.

### Experiments

We infected fifty C57/Bl6 10–12 week old female mice with 10^5^ *Plasmodium chabaudi*, genotype ER_710_ parasitised red blood cells (RBC) at ring stage by intraperitoneal injection. The *P*. *chabaudi* parasites used have not been previously exposed to, and were sensitive to, pyrimethamine. Pyrimethamine treatment at doses of 0 (*N* = 6 infections), 0.5 (*N* = 9), 1 (*N* = 9), 2 (*N* = 9), 10 (*N* = 9) or 25 mg/kg (*N* = 8), dissolved in 20 μL DMSO, was administered by intraperitoneal injection at 2pm GMT on day 11 post infection (PI). Red blood cell (RBC), asexual stage and gametocyte densities at the time of treatment did not differ significantly across treatment groups (RBC: *F*_5,33_ = 0.258, *P* = 0.93; asexual: *F*_5,33_ = 0.917, *P* = 0.48; gametocytes *F*_5,33_ = 0.859, *P* = 0.52). We quantified RBC densities by flow cytometry and parasite dynamics by quantitative PCR (qPCR, see ‘Quantifying parasite densities’ in S1 Text), daily from day 9–18 PI [53, 66].

We also carried out analyses of previously published data for genotypes CWvir (experiment 3 in [66], *N =* 20 infections) and AS and AJ (*N* = 25 infections [25]) to investigate the influences of state and RBC on conversion rates. Briefly, the experiment using CWvir exposed parasites to 0, 4, 8, 12 or 20 mg/kg pyrimethamine on day 5 PI (*N* = 4 infections per dose). RBC, asexual stage, gametocyte densities and the proportion of asexual stages killed (“state”) on day 11 PI are presented in S4 Table for comparison to the main experiment with ER and treatment on day 11 PI. The experiment using AS and AJ exposed parasites to within-host competition with 1 or 2 genetically distinct genotypes for the duration of infections. The AS and AJ data included parasites in single genotype infections (*N* = 5 AJ, *N* = 5 AS) and experiencing within-host competition in mixed-infections (*N* = 10 AJ and *N* = 5 AS). We combine AS and AJ data to achieve sufficient power for an analysis covering a broad range for variation in state. For both additional datasets, RBC were quantified using flow cytometry and gametocyte densities using RT-qPCR. Asexuals were quantified using qPCR for AS and AJ (see ‘Quantifying parasite densities’ in S1 Text) and by microscopy for CWvir.

To explore how conversion rates relate to RBC dynamics independently of changes in state, we analysed two further experiments that manipulated RBC densities / age structure by administering erythropoietin (EPO) [26] or phenylhydrazine (PHZ) [27]. For the EPO experiment [26], 15 C57Bl/6 mice were infected with *P*. *chabaudi* genotype ER, and treated with 0.1mL of 100U/L EPO on days 3–7 post infection (*N* = 8), or placebo (*N* = 7). EPO treatment increased reticulocyte densities, but did not significantly alter asexual parasite densities (i.e. no effect on state) [26]. Conversion rates were determined based on microscopy counts of asexual parasites and gametocytes. One EPO-treated mouse was excluded from the analyses because the residuals showed a significant relationship to natural logged densities of gametocytes (see ‘Calculating conversion rate’ in S1 Text). In the PHZ experiment [27], C57Bl/6 mice were treated with PHZ at doses of placebo (n = 22), 30 (n = 23) or 120mg/kg (n = 23) 4 days prior to infection with *P*. *chabaudi* genotype AS, AJ, CR or ER. Phenylhydrazine treatment resulted in a dose-dependent influx of reticulocytes by the time infections were initiated, but there was no significant difference in asexual densities between genotypes or PHZ treatments (i.e. no effect on state) [27]. Because the data series was too short to apply the Greischar method [54] for calculating conversion, we used the gametocyte densities on day 3 PI divided by asexual densities on day 1 PI to approximate conversion rates. This approximation can only be used reliably when accumulation of gametocytes from previous cohorts is minimal and independent of treatment group, as is the case at this early time point.

All experiments were carried out in accordance with the UK Animals Scientific Procedures Act 1986 and have been subject to ethical review and approved by the Home Office and the University of Edinburgh.

### Data analysis

All statistical analyses were carried out with R version 3.1.3 package mgcv 1.8–6 (The R-Foundation, Vienna, Austria). We minimised nested models using maximum likelihood deletion tests and compared non-nested models with AIC. First, we used generalised linear models to confirm that there were no between-group differences in densities of RBCs, asexual stages or gametocytes at the time of administering drug treatment to ER infections. Second, to analyse conversion rates on the day of drug treatment we used generalised additive models with a normal error distribution, after square root transformation to meet assumptions of homogeneity of variance. To determine the best measure for state, we constructed generalised additive models including the proportion or absolute number of asexual parasites killed for the day before, or the day during which, parasites commit to sexual reproduction, and asexual stage density on the day of sexual commitment. We used AIC to select the model explaining the most variance in conversion rate (S1 Table). We then extended these models to explore whether RBC resources also correlate with conversion rates. Third, to determine how the observed reaction norm for conversion rate against state impacts on between-host transmission, we multiplied conversion rate with asexual stage density. We converted these predicted gametocyte densities into probabilities of transmission (fitness) according to Eq 4 above, assuming all other factor that influence transmission (a.o. mosquito biting rate, host immunity, mosquito susceptibility) are equal [61]. Although time-of-day may affect transmission efficacy [81, 82], host, parasite and mosquito time-of-day have been controlled for in our experiment thus allowing for comparison of transmission probability between groups. The proportional change in transmission probability resulting from changing vs. not changing conversion, is estimated as transmission probabilities of drug treated groups relative to that of the untreated groups. Fourth, we estimated the impact of the predicted reaction norm of conversion rate against state for within-host survival as the number of asexual parasites from the focal cohort that contribute to asexual replication (i.e. “1 –conversion rate”) before drug killing occurs and the survivors undergo schizogony. The change in asexual density due to changing conversion is presented as the difference in asexual density of the treated and the untreated groups. Finally, we used generalised additive models, as described for ER, to analyze previously published data for CWvir [66] and AS and AJ [25]. In contrast to the analysis of ER data, we defined state as replication rate to capture the much greater range of changes in asexual density (i.e. from parasites tripling in every replication cycle to severe decline) than in our main experiment. For CWvir it was also necessary to control for day PI and differences in RBC densities between treatment groups at the start of data collection. For the EPO [26] and phenylhydrazine experiments [27], analyses were performed as described above, with the exception that we test if conversion rates depend on changes in RBC resources, not state. Additionally, for the EPO experiment, generalised additive mixed models were used, including mouse as a random effect.

## Supporting information

S1 Fig Infection dynamics resulting from predicted optimal conversion rate strategies. (*A*) The dynamics of uninfected RBCs, (*B*) infected RBCs and (*C*) gametocytes, for the optimal strategies in untreated infections (green: 0 mg/kg), and during low dose (purple: 5 mg/kg) or high dose (blue: 15 mg/kg) drug treatment. All infections follow the same patterns (black) before drug treatment is given on day 11 PI (indicated with vertical grey bar).
(TIF)

S2 Fig Infection dynamics for genotype ER asexual stages and gametocyte densities in the main experiment. Pyrimethamine drug treatment at day 11 post infection (PI) reduces asexual parasite densities (closed symbols) and affects gametocyte densities (open symbols). All doses are subcurative (*B-F*) and dynamics of untreated control infections (*A*) are as expected, i.e. declining because infections are in the post-peak phase. Mean ± SEM plotted for each dose-specific treatment group (0 mg/kg (*A*); 0.5 mg/kg (*B*); 1 mg/kg (*C*); 2 mg/kg (*D*); 10 mg/kg (*E*); 25 mg/kg (*F*)). Note that conversion rates cannot easily be deduced from comparing gametocyte dynamics during infections because, for example, gametocytes of multiple cohorts can overlap. Details about how the method of inference we use to estimate converison [54] overcomes these issues can be found in S1 Text and a summary of conversion rates related to these parasite dynamics on day 11 PI is presented in Fig 3.
(TIF)

S3 Fig Non-linear reaction norm for conversion rate as a function of the absolute number of asexual stages killed by antimalarial drugs. The predicted pattern for converison is recovered when the density of asexuals lost is used as a proxy for state. Specifically, ER parasites reduce conversion rates when less than 1.5 x10^5^ asexual stages are killed and increase their conversion when larger numbers of parasites are killed. Solid line illustrates the predicted pattern (± SEM, shaded area) from a generalised additive model. Note, the proportion of asexual stages killed correlates more closely with conversion rates than the absolute number killed (Main text Fig 3*B*, S1 Table).
(TIF)

S1 Table Statistical model selection to identify proxies for “state” that correlate with conversion rate (CR). (DOCX)

S2 Table Statistical model selection to identify putative cues for conversion rate (CR) decisions. (DOCX)

S3 Table Parameter values used in the mathematical within-host model of state-dependent conversion rates. (DOCX)

S4 Table Comparison of infection parameters for the main experiment following *P*. *chabaudi* genotype ER treated on day 11 PI and the day 11 PI for the additional data analysed for CWvir (treated on day 5 PI). (DOCX)

S1 Text Additional materials and methods. (DOCX)

S2 Text SI references. (DOCX)
