# Supplementary figures and images for: Adaptive plasticity in the gametocyte conversion rate of malaria parasites
Source: PLoS Pathog. 2018 Nov 14;14(11):e1007371. doi: 10.1371/journal.ppat.1007371 (PMC6261640; doi:10.1371/journal.ppat.1007371)

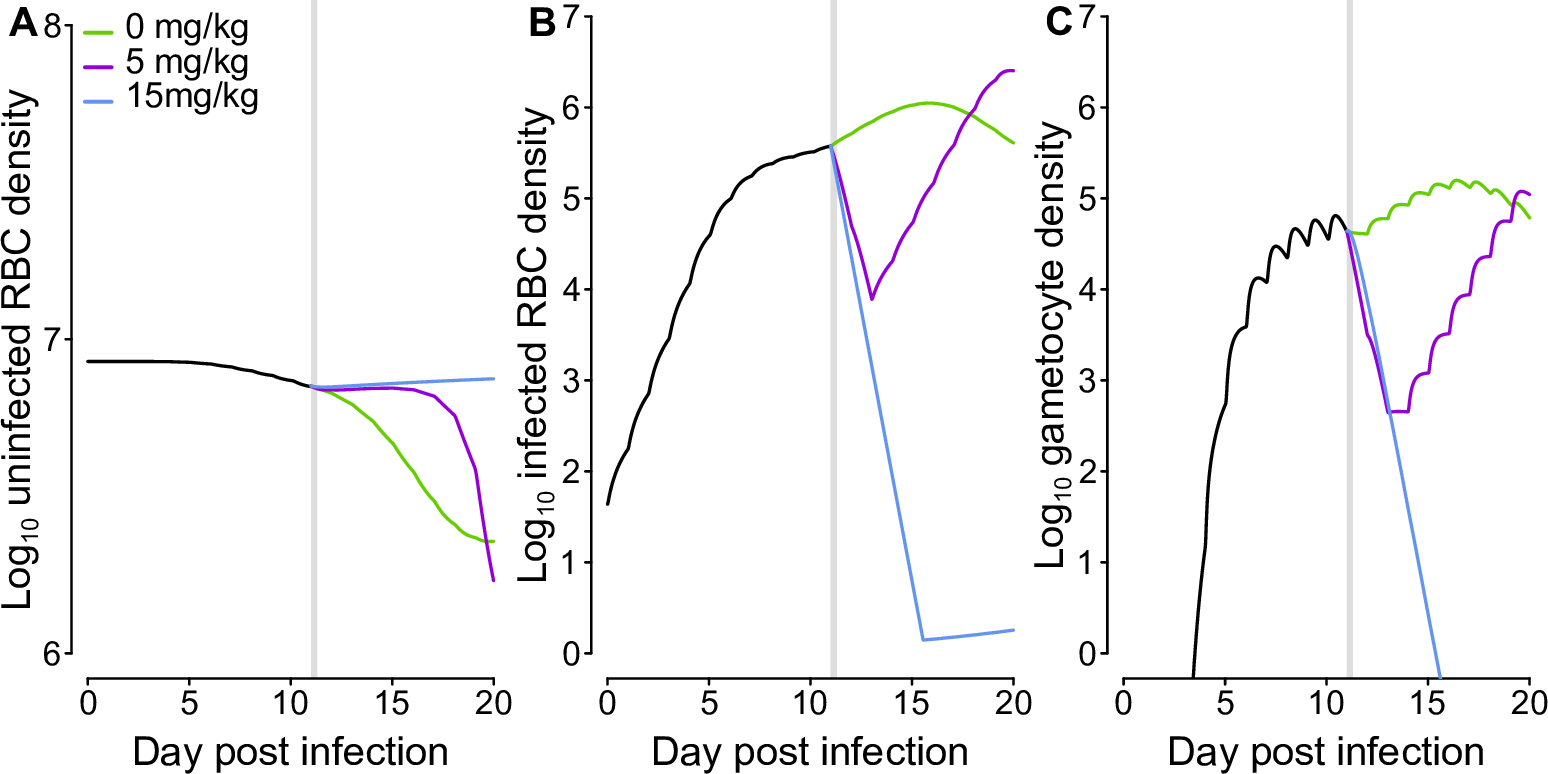

Supplement: S1 Fig — (A) The dynamics of uninfected RBCs, (B) infected RBCs and (C) gametocytes, for the optimal strategies in untreated infections (green: 0 mg/kg), and during low dose (purple: 5 mg/kg) or high dose (blue: 15 mg/kg) drug treatment. All infections follow the same patterns (black) before drug treatment is given on day 11 PI (indicated with vertical grey bar). (TIF) [file ppat.1007371.s001.tif]

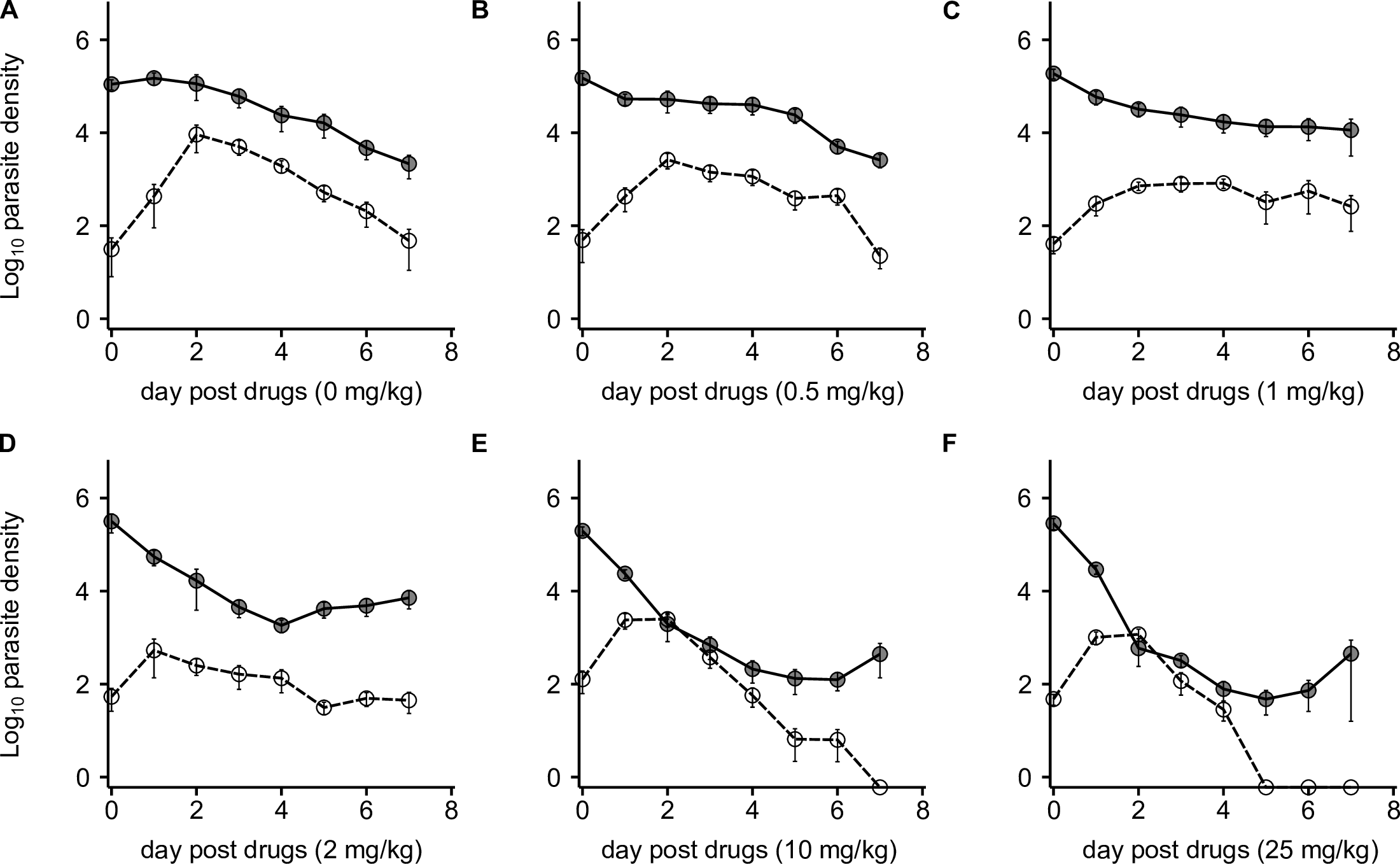

Supplement: S2 Fig — Pyrimethamine drug treatment at day 11 post infection (PI) reduces asexual parasite densities (closed symbols) and affects gametocyte densities (open symbols). All doses are subcurative (B-F) and dynamics of untreated control infections (A) are as expected, i.e. declining because infections are in the post-peak phase. Mean ± SEM plotted for each dose-specific treatment group (0 mg/kg (A); 0.5 mg/kg (B); 1 mg/kg (C); 2 mg/kg (D); 10 mg/kg (E); 25 mg/kg (F)). Note that conversion rates cannot easily be deduced from comparing gametocyte dynamics during infections because, for example, gametocytes of multiple cohorts can overlap. Details about how the method of inference we use to estimate converison [54] overcomes these issues can be found in S1 Text and a summary of conversion rates related to these parasite dynamics on day 11 PI is presented in Fig 3. (TIF) [file ppat.1007371.s002.tif]

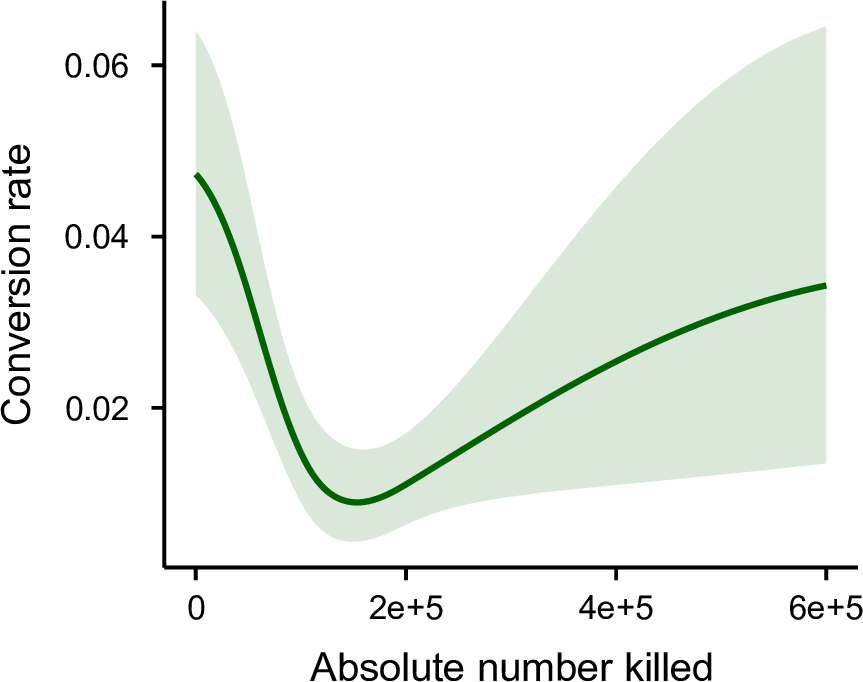

Supplement: S3 Fig — The predicted pattern for converison is recovered when the density of asexuals lost is used as a proxy for state. Specifically, ER parasites reduce conversion rates when less than 1.5 x10^5^ asexual stages are killed and increase their conversion when larger numbers of parasites are killed. Solid line illustrates the predicted pattern (± SEM, shaded area) from a generalised additive model. Note, the proportion of asexual stages killed correlates more closely with conversion rates than the absolute number killed (Main text Fig 3B, S1 Table). (TIF) [file ppat.1007371.s003.tif]
