# Supplementary material for: Adaptive plasticity in the gametocyte conversion rate of malaria parasites
Source: PLoS Pathog. 2018 Nov 14;14(11):e1007371. doi: 10.1371/journal.ppat.1007371 (PMC6261640; doi:10.1371/journal.ppat.1007371)
Supplement: S1 Table — (DOCX) [file ppat.1007371.s004.docx]

**S1 Table. Statistical model selection to identify proxies for “state” that correlate with conversion rate (CR)**

|  | AIC  gam | adj. r^2^ | ΔAIC  linear | ΔAIC  quadratic | ΔAIC  cubic |
| --- | --- | --- | --- | --- | --- |
| **CR ~ proportion asexuals lost** | **-74.31** | **0.331** | **12.41** | **2.68** | **3.02** |
| CR ~ absolute number asexuals lost | -66.00 | 0.171 | 3.35 | 1.24 | 2.71 |

**Differences in AIC (ΔAIC) suggest that generalized additive models (gam) without restrictions on the pattern for the state variable fit better than linear, quadratic and cubic models. Further, the proportion of parasites lost, relative to untreated infections, correlates more closely with conversion rates than the number of parasites lost.**
