# Supplementary material for: Adaptive plasticity in the gametocyte conversion rate of malaria parasites
Source: PLoS Pathog. 2018 Nov 14;14(11):e1007371. doi: 10.1371/journal.ppat.1007371 (PMC6261640; doi:10.1371/journal.ppat.1007371)
Supplement: S2 Table — (DOCX) [file ppat.1007371.s005.docx]

**S2 Table. Statistical model selection to identify putative cues for conversion rate (CR) decisions**

|  | RBC factor | *P* -value | AIC | adj. r^2^ |
| --- | --- | --- | --- | --- |
| CR ~ proportion asexuals killed | - | - | -74.31 | 0.331 |
| (1) | RBC | 0.012 | -78.89 | 0.418 |
|  | state* RBC | 0.125 |  |  |
| **(2)** | **state*RBC** | **0.003** | **-92.20** | **0.614** |
| (3) | RBC | **0.196** |  |  |
|  | state* RBC | **0.184** |  |  |

Three statistical models were fitted to include the following RBC metrics as main effects and in interactions with state**: (1) The absolute density of RBC midway through the cycle of the decision-making cohort. (2) The difference between RBC densities experienced by the decision-making cohort and its parental cohort. (3) The absolute density of RBC midway through the cycle of the parental cohort. The minimal models and variables removed during model minimization are shown and those remaining are in bold.**
