# Supplementary material for: Adaptive plasticity in the gametocyte conversion rate of malaria parasites
Source: PLoS Pathog. 2018 Nov 14;14(11):e1007371. doi: 10.1371/journal.ppat.1007371 (PMC6261640; doi:10.1371/journal.ppat.1007371)
Supplement: S3 Table — (DOCX) [file ppat.1007371.s006.docx]

**S3 Table. Parameter values used in the mathematical within-host model of state-dependent conversion rates.**

| Parameter | Description | Value or range | References |
| --- | --- | --- | --- |
| *R** | Red blood cell (RBC) density of a healthy mouse | 8.5x10^6^ RBCs/μL | [76] |
| λ | Maximum rate of RBC production | 3.7x10^5^ RBCs/μL | [76] |
| *μ* | Death rate of RBCs | 0.025/day | [77] |
| *μ_M_* | Death rate of merozoites | 48/day | [78] |
| *μ_G_* | Death rate of gametocytes | 4/day | [28] |
| *μ_d_* | Drug-induced death rate of infected cells | 2.81/day | [74] |
| *p* | Maximum rate of invasion per merozoite | 4x10^-6^/day | [75] |
| *α* | Parasite cell cycle duration | 1 day | [79] |
| *β* | Burst size | 10 merozoites | [78] |
| *d* | Drug dose | 0-15 mg/kg |  |
| *I*_0_ | Initial dose of infected RBCs | 44/μl (~10^4^/mouse) | [56] |
| *R*_11_ | Density of susceptible RBCs before drug treatment (day 11) | 7098394/μL | Predicted from model |
| *I*_11_ | Density of infected RBCs before drug treatment (day 11) | 375259/μL | Predicted from model |
| *M*_11_ | Density of merozoites before drug treatment (day 11) | 8462/μL | Predicted from model |
| *G*_11_ | Density of gametocytes before drug treatment (day 11) | 43520/μL | Predicted from model |
|  | Coefficients for cubic spline defining optimal conversion, $c(t),$ in the absence of drugs (up to day 11) | 7.794506,  -15.93132,  -4.253001,  -9.835005 | [56] |
