# Supplementary material for: Adaptive plasticity in the gametocyte conversion rate of malaria parasites
Source: PLoS Pathog. 2018 Nov 14;14(11):e1007371. doi: 10.1371/journal.ppat.1007371 (PMC6261640; doi:10.1371/journal.ppat.1007371)
Supplement: S4 Table — (DOCX) [file ppat.1007371.s007.docx]

**S4 Table. Comparison of infection parameters for the main experiment following *P. chabaudi* genotype ER treated on day 11 PI and the day 11 PI for the additional data analysed for CWvir (treated on day 5 PI).**

| **DOSE** | **RBC** | | **ASEX** | | **GCT** | | **STATE** | |
| --- | --- | --- | --- | --- | --- | --- | --- | --- |
|  | **ER** | **CWvir** | **ER** | **CWvir** | **ER** | **CWvir** | **ER** | **CWvir** |
| 0 | 2.6±0.4 | 1.5±0.2 | 5.0±0.1 | 5.2±0.1 | 1.5±0.6 | 1.9±0.1 | 0.119±0.257 | 0.330±0.120 |
| 0.5 | 2.7±0.4 |  | 5.1±0.1 |  | 1.7±0.7 |  | 0.591±0.222 |  |
| 1 | 3.0±0.4­­­ |  | 5.3±0.2 |  | 1.4±0.6 |  | 0.754±0.109 |  |
| 2 | 2.7±0.3 |  | 5.5±0.3 |  | 1.8±0.3 |  | 0.886±0.086 |  |
| 4 |  | 3.5±0.6 |  | 6.0±0.1 |  | 4.1±0.4 |  | 0.399±0.068 |
| 8 |  | 7.0±0.6 |  | 5.6±0.5 |  | 3.8±0.9 |  | -1.958±1.023 |
| 10 | 2.9±0.3 |  | 5.4±0.1 |  | 2.2±0.4 |  | 0.987±0.008 |  |
| 12 |  | 7.2±0.3 |  | 4.7±0.1 |  | 2.5±0.3 |  | -5.688±1.527 |
| 20 |  | 7.3±0.5 |  | 3.6±0.6 |  | 1.1±0.1 |  | -4.861±3.100 |
| 25 | 2.6±0.2 |  | 5.5±0.1 |  | 1.7±0.1 |  | 0.998±0.002 |  |

RBC: RBC*10^9^/mL; ASEX: ^10^log asexual parasites densities/μL; STATE: proportion asexual parasites lost between day 11 and 12 PI (thus, negative values represent positive parasite replication); GCT: ^10^log gametocyte densities/μL.
