# Supplementary material for: Adaptive plasticity in the gametocyte conversion rate of malaria parasites
Source: PLoS Pathog. 2018 Nov 14;14(11):e1007371. doi: 10.1371/journal.ppat.1007371 (PMC6261640; doi:10.1371/journal.ppat.1007371)
Supplement: S1 Text — (DOCX) [file ppat.1007371.s008.docx]

**S1 file. Additional Materials and Methods**

**Calculating conversion rate**

Calculating conversion rate requires the quantification of a cohort of asexual stages at the time of commitment to gametocytes, as well as quantification of gametocytes formed by that cohort of asexuals. Because sexual stages cannot be identified (nor quantified), at the exact time of commitment, conversion rates are always estimated [3, 24 36]*.* Previous methods make assumptions (fixed conversion during maturation of sexual stages; equal death rates for asexuals and gametocytes; short survival of gametocytes and thus, non-overlapping cohorts of gametocytes) that are unrealistic for much of the duration of untreated infections and certainly during drug treatment [3]. A recent method of inference developed by Greischar et al [54] is not conditional on such assumptions and enables a more robust estimation of conversion rates in conditions where parasite densities decline. In this method, daily counts of gametocytes for time-series spanning at least 7 days, are linked to the cohort of asexual parasites from which the sexual stages originate. Specifically, Greischar’s method [54] estimates gametocyte densities throughout the infections based on the starting density of gametocytes, and the dynamics of asexual stages and RBC during infections. It estimates both the gametocyte half-life (from a range of 0.1-41hrs; [26]) and conversion rate by comparing candidate splines (constant, linear, parabolic, cubic or cubic with an interior knot). Since the method [54] uses forward selection, it only selects more complicated patterns for conversion if they provide a substantially better fit to the data.

To relate gametocytes to their parental cohort of asexuals, the earliest age of detection by the gametocyte-specific PCHAS_0620900 RT-qPCR assay was determined for data from the main experiment (using ER). Pyrimethamine kills asexually replicating stages but not gametocytes [36, 83]. Drugs can therefore only affect gametocyte densities by reducing the pool of asexuals that can commit and/or parasites adjusting conversion rate. The first asexual cohort for which either of these can occur is the cohort of parasites exposed to pyrimethamine on day 11 PI. Gametocyte densities do not differ significantly between treatment groups until day 13 PI (*F*_5,43_ = 2.98, *P* = 0.02). These gametocytes largely originate from the day 11 PI cohort of asexual stages. Due to daily sampling from 09:00 to 11:00 GMT, and schizonts bursting from 00:00 to 03:00 GMT to release asexual- or gametocyte-committed progeny [84], gametocytes are first detected by our assay 30-35 hours after bursting from a sexually committed schizont, approximately 48 hrs after the detection of the asexual ring stages that the gametocytes originate from. This is in line with previous observations that sexual stages at maturation are approximately 54 hours old [28], which includes the 24 hours required for the development of the asexuals that commit to producing them*.*

We extended Greischar et al’s [54] method to test whether the model fits the data by comparing the fit of predicted versus measured gametocyte densities. Infections for which the residuals showed a significant relationship to natural logged densities of gametocytes were excluded; these seem to result from the error structure used for counts of gametocytes, which was defined using data with much higher gametocyte densities [74]. This affected *N*=0, 1, 3, 0, 2 and 0 infections respectively, for doses 0, 0.5, 1, 2, 10 and 25 mg/kg in the ER dataset; *N* =0, 0, 1, 1 and 1 respectively for doses 0, 4, 8, 12 and 20 mg/kg in the CWvir dataset; and *N* =0, 2, 1 and 2 for AS single, AJ single, AS mixed and AJ mixed genotype infections. Exclusions were not related to treatment groups. Furthermore, infections for which less than four of the five candidate splines could be fitted (i.e. time series require at least seven subsequent observations) were excluded from the analyses. For ER, this included *N*=0, 1, 1, 1, 1 and 1 respectively for doses of 0, 0.5, 1, 2, 10 and 25 mg/kg; none were excluded for CWvir or AS /AJ single infections; *N*=1 were excluded for AS mixed and *N*=2 for AJ mixed infections. Exclusions were not related to treatment groups.

**Quantifying parasite densities**

Total parasites and sexual stages were quantified by quantitative PCR using respectively DNA and cDNA, and primers based on the sexual stage-specific expressed gene PCHAS_0620900, previously named PC302249.00.0 [53]. Asexual parasites were quantified by subtracting the gametocyte counts from the total parasite counts. For DNA extraction, 5 μL blood samples were mixed with 150 μL citrate saline (0.85 % w/v NaCl, 1.5% w/v trisodium citrate dihydrate) to prevent clotting, centrifuged, and after removal of the supernatant the blood pellets were frozen at -70°C. Ten μL blood samples for RNA extraction were mixed with 20 μL RNAlater® and frozen at -70°C. DNA and RNA were extracted using the semi-automatic Kingfisher Flex Magnetic Particle Processor using the MagMax 96-DNA multisample kit for DNA and the MagMax 96-Total RNA Isolation kit for RNA (Thermo Fisher Scientific). Extraction protocols for both DNA and RNA have been adjusted from the standard protocols 4413021DWblood and AM1830DW to improve recovery [27]. RNA extracts were diluted 1:4 to prevent downstream effects of inhibitors, and cDNA was produced by reverse transcriptase PCR according to manufacturer’s protocol (High-Capacity cDNA Reverse Transcription Kit, Thermo Fisher Scientific).
