# Supplementary material for: Adaptive plasticity in the gametocyte conversion rate of malaria parasites
Source: PLoS Pathog. 2018 Nov 14;14(11):e1007371. doi: 10.1371/journal.ppat.1007371 (PMC6261640; doi:10.1371/journal.ppat.1007371)
Supplement: S2 Text — (DOCX) [file ppat.1007371.s009.docx]

**References**

83. Duffy S, Avery VM. Identification of inhibitors of *Plasmodium falciparum* gametocyte development. *Malar J* 2013;12(1):408.

84. O'Donnell AJ, Schneider P, McWatters HG, Reece SE. Fitness costs of disrupting circadian rhythms in malaria parasites. *Proc R Soc Lond Ser B*. 2011;278(1717):2429-2436.
